# IFN-γ/TNF-α Synergism Induces Pro-Inflammatory Cytokine and Chemokine Production by In Vitro Canine Keratinocytes

**DOI:** 10.3390/vetsci12010055

**Published:** 2025-01-14

**Authors:** Kyungsook Jung, Ji-Yeong Ku, Je-Seong Kwon, Gayeon Won, Hakyoung Yoon, Sang-Ik Oh, Mi Hye Kim, Chongchan Kim, Ji-Seon Yoon

**Affiliations:** 1 Functional Biomaterials Research Center, Korea Research Institute of Bioscience and Biotechnology, Jeongeup 56212, Republic of Korea; jungks@kribb.re.kr; 2 Biosafety Research Institute and College of Veterinary Medicine, Jeonbuk National University, Iksan 54896, Republic of Korea; as05064@jbnu.ac.kr (J.-Y.K.); magick4501@naver.com (J.-S.K.); gywon@jbnu.ac.kr (G.W.); hyyoon@jbnu.ac.kr (H.Y.); sioh@jbnu.ac.kr (S.-I.O.); 3 College of Korean Medicine, Woosuk University, Wanju 55338, Republic of Korea; kimmh526@woosuk.ac.kr; 4 Korea Thumb Vet Co., Ltd., 470-15 Seonhwa-ro, Iksan 54631, Republic of Korea; jongchan1983@hanmail.net

**Keywords:** canine, keratinocyte, interferon γ, tumor necrosis factor α, synergistic effects, pro-inflammatory cytokine

## Abstract

Activated keratinocytes play a crucial role in skin inflammation through the production of multiple inflammatory mediators; however, little is known about cytokine secretion by activated keratinocytes in dogs. This study aimed to investigate the effects of the Th1 and Th2 types of cytokines on the production of keratinocyte-derived inflammatory mediators. Canine progenitor epidermal keratinocytes (CPEKs) were incubated with canine recombinant IL-4, IL-13, an IL4/IL13 mixture, IFN-γ, TNF-α, or an IFN-γ/TNF-α mixture for 24 h following 100% confluency. Culture supernatants were analyzed for cytokine concentration, including chemokine ligand (CXCL) 8, IL-10, IL-6, IL-1ß, IL-12, and chemokine ligand 2 (CCL2) by enzyme-linked immunosorbent assay. CPEKs incubated with the IFN-γ/TNF-α mixture showed significantly increased IL-6 concentration. In addition, significantly increased concentrations of CXCL8 were detected in CPEKs incubated with TNF-α and with the IFN-γ/TNF-α mixture. CCL2 concentrations increased in cells incubated with IFN-γ, TNF-α, and the IFN-γ/TNF-α mixture. The IFN-γ/TNF-α mixture synergistically enhanced CCL2 production. Dose-dependent elevations were also observed in IL-6 in response to the IFN-γ/TNF-α mixture, and in CCL2 in response to IFN-γ, TNF-α, and the IFN-γ/TNF-α mixture. These findings indicate that IFN-γ and TNF-α synergistically increase pro-inflammatory cytokines and chemokines secreted by canine keratinocytes. This in vitro culture system could be useful to investigate cytokine-mediated crosstalk between keratinocytes and immune cells and new therapeutic strategies for keratinocyte-mediated inflammatory skin diseases.

## Simple Summary

The present study investigated the effects of Th2- or Th1-type cytokines on the production of keratinocyte-derived inflammatory mediators. Canine progenitor epidermal keratinocytes (CPEKs) were incubated with IL-4, IL-13, an IL4/IL13 mixture, IFN-γ, TNF-α, or an IFN-γ/TNF-α mixture. After 24 h, levels of chemokine ligand (CXCL) 8, IL-10, IL-6, IL-1ß, IL-12, and chemokine ligand 2 (CCL2) were analyzed by enzyme-linked immunosorbent assay (ELISA). The IFN-γ/TNF-α mixture synergistically increased the IL-6 concentration in CPEKs. In addition, CXCL8 concentrations were significantly increased in CPEKs incubated with TNF-α and the IFN-γ/TNF-α mixture. Increased concentrations of CCL2 were observed after treatment with IFN-γ, TNF-α, and the IFN-γ/TNF-α mixture, and this mixture synergistically increased CCL2 production. Furthermore, IL-6 and CCL2 concentrations increased in a dose-dependent manner. These findings suggest that the Th1 cytokines IFN-γ and TNF-α synergistically increase pro-inflammatory cytokines and chemokines in canine keratinocytes and that these cytokines might aggravate skin inflammation.

## 1. Introduction

Keratinocytes, the predominant cell type in the epidermis, contribute to maintaining tissue homeostasis and skin barrier function. In addition, keratinocytes play a critical role in the cutaneous immune system, particularly in inflammatory skin diseases, including atopic dermatitis (AD) [1,2,3]. Accordingly, epidermal keratinocytes can be activated by exogenous factors (allergens or microbes) and by endogenous factors produced by immune cells, releasing various inflammatory mediators to induce further immunoreactions [1,2,3,4]. Keratinocytes express a large variety of cytokines (interleukins, IL) and chemokines (chemokine ligands with a C-C and a C-X-C motif), which secrete pro-inflammatory signals to recruit immune cells to the site of injury, infection, or inflammation [4].

In vitro models mimicking the features of skin inflammation in humans by keratinocyte activation using cytokine cocktails have been reported [5,6,7,8]. These cytokine cocktails include the Th2-type IL-4 and IL-13, the Th1-type interferon γ (IFN-γ) and tumor necrosis factor α (TNF-α), and the Th17-type IL-17, which have been reported to be involved in skin inflammation related to human AD or psoriasis [5,6,7,8]. Accordingly, supplementation of epidermal keratinocytes with these cytokines induces the production of keratinocyte-derived inflammatory mediators such as IL-1β, IL-6, IL-23, chemokine ligand (CCL) 5, and chemokine ligand (CXCL) 8 [5,6,7,8]. Among them, the mixture of IFN-γ and TNF-α synergistically induces an inflammatory response involving the secretion of keratinocyte-derived cytokines and inflammatory cell death [6,9]. Cultures of a single cell type, such as keratinocytes, could be the simplest in vitro models to investigate the molecular pathogenesis involved in the etiological processes of epidermal inflammatory diseases.

Similarly to human AD, several cytokines (including IL-4, IL-13, IL-5, IL-13, IFN-γ, and TNF-α) have been reported to be upregulated in spontaneous or experimentally induced AD in dogs [10,11]. However, little is known about the effects of these Th2- or Th1-type cytokines on the secretion of inflammatory mediators by activated keratinocytes in dogs. Investigation of the production of inflammatory mediators in activated keratinocytes by cytokine production might provide a deeper understanding of cytokine-mediated crosstalk between keratinocytes and immune cells in canine AD. In addition, the development of an in vitro model mimicking features of skin inflammation in dogs could be a useful tool to investigate new therapeutic strategies in the future. Therefore, the present study aimed to investigate the effect of Th2-type or Th1-type cytokines on the production of keratinocyte-derived inflammatory mediators, and to develop an in vitro culture system to investigate cytokine-mediated crosstalk by keratinocytes in inflammatory canine skin diseases.

## 2. Materials and Methods

### 2.1. Chemicals and Reagents

To stimulate canine keratinocytes, Th2 cytokines (IL-4 and IL-13) and Th1 cytokines (IFN-γ and TNF-α), reported to be elevated in canine AD, were selected in this study. Canine recombinant IL-4, IL-13, IFN-γ, and TNF-α were purchased from R&D systems (Minneapolis, MN, USA).

### 2.2. Cell Culture

Canine progenitor epidermal keratinocytes (CPEKs) were purchased from CellnTec (Bern, Switzerland). Cells seeded on a 10-centimeter dish were cultured using CnT-09 Canine Epithelial Proliferation Medium (CellnTec). The culture media was incubated at 37 °C in a cell incubator with 5% CO_2_. To investigate cytokine production by different cytokine stimulators, CPEKs were seeded on 24-well plates (30,000 cells/well) and incubated with 30 ng/mL of canine recombinant IL-4, IL-13, an IL-4/IL-13 mixture, IFN-γ, TNF-α, and an IFN-γ/TNF-α mixture for 24 h. As our preliminary experiments showed an increase in cytokine concentrations in response to 30 ng/mL of the IFN-γ/TNF-α mixture compared to 10 ng/mL, and no significant differences between a 24-h and a 48-h incubation, we selected 30 ng/mL as the stimulus dosage, with 24 h of treatment. Additionally, to investigate dose-dependent effects on cytokine productions, CPEKs were incubated with increasing concentrations of IFN-γ (10, 30, 60, and 90 ng/mL), TNF-α (10, 30, 60, and 90 ng/mL), or the IFN-γ/TNF-α mixture (10, 30, 60, and 90 ng/mL each) for 24 h at 37 °C. Culture supernatants were then collected and preserved at −20 °C for immunoassay analysis. Five independent experiments were performed in each case.

### 2.3. Immunoassay

ELISAs for the keratinocyte-derived cytokines IL-10, IL-1ß, IL-12, IL-8/C-X-C motif CXCL8, and the C-C motif chemokine ligand 2 (CCL2) were performed. Canine IL-6, IL-10, IL-1ß, IL-12, CXCL8, and CCL2 DuoSet ELISA kits (R&D systems) were used according to the manufacturer’s instructions. Accordingly, 100 µL of the cell culture supernatant was used for the analysis of IL-6 concentrations. Following the overexpressions of samples in CXCL8 and CCL2 concentrations, the samples were diluted to the proper concentration for detection. Samples were analyzed in duplicate.

### 2.4. Statistical Analysist

Statistical analyses were performed using the SPSS Statistics 29 software package (Version 29.0. Armonk, NY, USA, IBM Corp.). The normality of data was analyzed by the Shapiro–Wilk test. If the *p*-value was less than 0.05, the data were considered as not being normally distributed. For the effect of different cytokine stimulators on cytokine production, each dataset was compared to that of the control group treated with vehicle, using the Mann–Whitney U test or an unpaired Student’s *t*-test, depending on the normality. In addition, dose-dependent effects of cytokines were compared by one-way analysis of variance (ANOVA) with Tukey’s Honestly Significant Difference (HSD) post hoc test or the Kruskal–Wallis test with Dunnett’s post -hoc test, depending on the normality. Data represents the mean of five independent experiments ± standard deviation. A *p*-value < 0.05 was considered statistically significant.

## 3. Results

### 3.1. Comparison of the Production of Keratinocyte-Derived Cytokines by Th2 or Th1 Cytokine Stimulators

**Figure 1:**
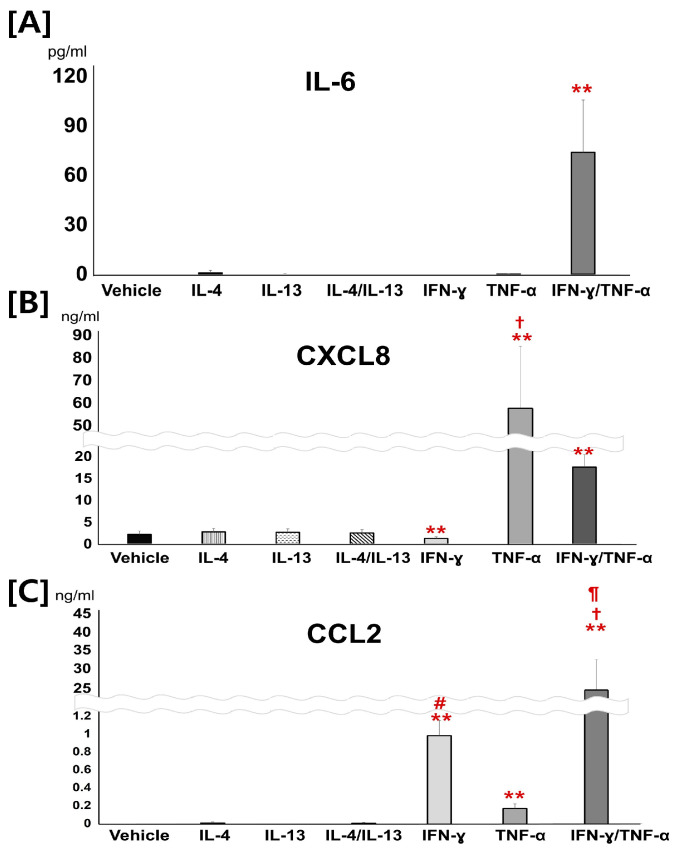
The effects of Th1 or Th2 cytokine stimulation on the production of IL-6, CXCL8, and CCL2 from canine progenitor epidermal keratinocytes (CPEKs). (**A**) CPEKs stimulated with an IFN-γ/TNF-α mixture showed increased IL-6 concentrations compared to vehicle (** *p* < 0.01, unpaired *t*-test). (**B**) Supernatants from CPEKs stimulated with TNF-α (** *p* < 0.01, Mann–Whitney U test) and the mixture of IFN-γ/TNF-α (** *p* < 0.01, unpaired *t*-test) showed significantly increased CXCL8 concentrations. CXCL8 levels were more elevated in cells stimulated by TNF-α than in those stimulated by the mixture of IFN-γ/TNF-α (+ *p* < 0.01, Mann–Whitney U test). The supernatants from CPEKs incubated with IFN-γ showed decreased CXCL8 concentrations (** *p* < 0.01, unpaired *t*-test). (**C**) The supernatants from CPEKs incubated with IFN-γ, TNF-α, and the IFN-γ/TNF-α mixture exhibited significantly increased CCL2 concentrations compared to vehicle (** *p* < 0.01, Mann–Whitney U test). Among them, those from CPEKs incubated with the IFN-γ/TNF-α mixture showed significantly increased CCL2 concentrations compared to those incubated with either IFN-γ or TNF-α (+ *p* < 0.01 TNF-α versus the IFN-γ/TNF-α mixture, ¶ *p* < 0.05, IFN-γ versus the IFN-γ/TNF-α mixture, Mann–Whitney U test). In addition, the supernatants of CPEKs incubated with IFN-γ showed significantly increased CCL2 concentrations compared to those from CPEKs incubated with TNF-α (# *p* < 0.01, unpaired *t*-test). Data represent the mean of five independent experiments ± standard deviation.

To investigate the effect of cytokine stimulators on the production of keratinocyte-derived cytokines, concentrations of IL-10, IL-1ß, IL-12, CXCL8, and CCL2 in CPEKs supplemented with 30 ng/mL of IL-4, IL-13, IL-4, and the IL-13 mixture, IFN-γ, TNF-α, and the IFN-γ/TNF-α mixture were compared with the vehicle. IL-6 was hardly detected in CPEKs incubated with vehicle, IL-4, IL-13, the IL4/IL13 mixture, IFN-γ, or TNF-α (Figure 1A). Meanwhile, CPEKs incubated with the IFN-γ/TNF-α mixture showed significantly increased IL-6 concentrations (70-fold) compared to the vehicle (Figure 1A). No significant differences were observed in CXCL8 levels synthesized by CPEKs stimulated with IL4, IL13, or the mixture of IL-4/IL-13 compared with the vehicle (Figure 1B). Furthermore, CPEKs stimulated with IFN-γ showed a significant decrease in CXCL8 production compared to the vehicle (Figure 1B). Meanwhile, CPEKs incubated with TNF-α and the mixture of IFN-γ/TNF-α showed significantly increased CXCL8 concentrations compared to the vehicle (Figure 1B). CXCL8 levels were more elevated in cultures stimulated by TNF-α than in those stimulated by the mixture of IFN-γ/TNF-α (Figure 1B). Furthermore, CCL2 was hardly detected in the supernatants of CPEKs treated with vehicle, IL-4, IL-13, or the IL-4/IL-13 mixture (Figure 1C). However, significantly increased CCL2 concentrations were detected in the supernatants of CPEKs supplemented with IFN-γ, TNF-α, and the IFN-γ/TNF-α mixture (Figure 1C). Among them, the mixture of IFN-γ/TNF-α synergistically enhanced CCL2 production when compared to single treatments with IFN-γ or TNF-α (Figure 1C). Meanwhile, the concentrations of IL-10, IL-1ß, and IL-12 were almost undetectable in any of the groups.

### 3.2. Investigation of Dose-Dependent Effects on the Production of IL6, CXCL8, and CCL2

**Figure 2:**
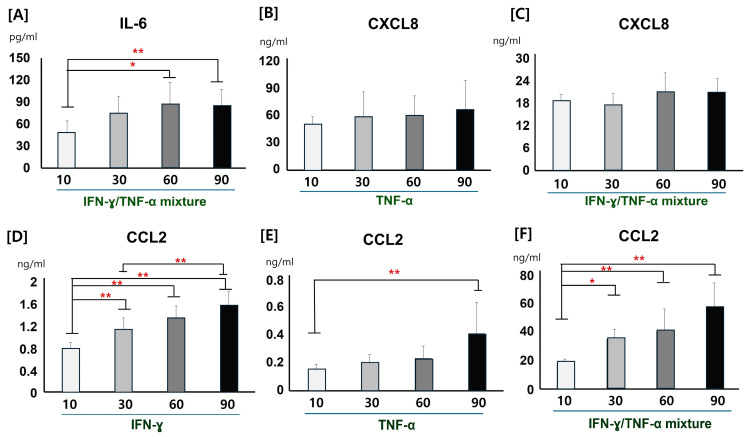
Dose-dependent effects on the production of IL-6, CXCL8, and CCL2 from canine progenitor epidermal keratinocytes (CPEKs). (**A**) CPEKs incubated with 60 and 90 ng/mL of the IFN-γ/TNF-α mixture showed significantly increased IL-6 concentrations in their culture medium compared to those treated with 10 ng/mL of the IFN-γ/TNF-α mixture (Kruskal–Wallis test with Dunnett’s test). (**B**) No significant differences in CXCL8 concentration were detected in response to treatment with different concentrations of TNF-α (10, 30, 60, and 90 ng/mL). (**C**) No significant differences in CXCL8 concentration were detected in response to treatment with different doses of the mixture of IFN-γ/TNF-α (10, 30, 60, or 90 ng/mL). (**D**) CPEKs incubated with 30, 60, or 90 ng/mL of IFN-γ showed significantly increased CCL2 concentrations in their culture medium compared to those treated with 10 ng/mL of IFN-γ (ANOVA with Tukey’s HSD). (**E**) CPEKs incubated with 90 ng/mL of TNF-α also showed significantly increased CCL2 concentrations in their culture medium than those treated with 10 ng/mL of TNF-α (ANOVA with Tukey’s HSD). (**F**) CPEKs incubated with 30, 60, or 90 ng/mL of the IFN-γ/TNF-α mixture showed significantly increased CCL2 concentrations in their culture medium than those treated with 10 ng/mL of the same mixture (Kruskal–Wallis test with Dunnett’s test). Data represent the mean of five independent experiments ± standard deviation. * *p* < 0.05, ** *p* < 0.01.

As significantly increased concentrations were observed for IL-6 in response to treatment with the mixture of IFN-γ/TNF-α, for CXCL8 in response to treatment with TNF-α and IFN-γ/TNF-α, and for CCL2 in response to treatment with IFN-γ, TNF-α, and IFN-γ/TNF-α, dose-dependent effects on these cytokine stimulators were also investigated. CPEKs incubated with 60 and 90 ng/mL of the IFN-γ/TNF-α mixture showed significantly increased IL-6 concentrations in their culture media compared to those incubated with 10 ng/mL of IFN-γ (Figure 2A). Meanwhile, CXCL8 concentrations showed no significant differences in response to different concentrations of TNF-α (10, 30, 60, or 90 ng/mL, Figure 2B) or the mixture of IFN-γ/TNF-α (10, 30, 60, or 90 ng/mL, Figure 2C). CPEKs incubated with 30, 60, or 90 ng/mL of IFN-γ showed significantly increased CCL2 concentrations in their culture medium compared to those treated with 10 ng/mL of IFN-γ (Figure 2D). CPEKs incubated with 90 ng/mL of TNF-α also showed significantly increased CCL2 concentrations compared to those treated with 10 ng/mL of TNF-α (Figure 2E). In addition, CPEKs incubated with 30, 60, and 90 ng/mL of the IFN-γ/TNF-α mixture showed significantly increased CCL2 concentrations in their culture medium compared to those treated with 10 ng/mL of the same mixture (Figure 2F).

## 4. Discussion

The present study evaluated the effects of single treatments with both Th1- or Th2-type cytokines or cytokine mixtures on the production of pro-inflammatory cytokines and chemokines by activated canine keratinocytes. It revealed that the Th1 cytokines IFN-γ and TNF-α induce the production of the pro-inflammatory cytokines and chemokines IL-6, CCL2, and CXCL8 by canine keratinocytes in vitro. However, no significant effects on IL-6, CCL2, or CXCL8 production were observed in CPEK cells incubated with the Th2 cytokines IL-4 and IL-13.

The increased CXCL8 concentrations observed in this study are similar to those reported in previous studies in humans [12,13]. A single treatment with TNF-α, as well as the mixture of IFN-γ/TNF-α, induced heightened CXCL8 concentrations in human keratinocytes [12,13]. Similarly, in a previous study using a canine keratinocyte cell line (MSCEK), keratinocytes were stimulated with IL-4, IL-13, IFN-γ, and TNF-α, and only keratinocytes stimulated with TNF-α showed significantly increased CXCL8 concentrations [14]. In addition, a previous study that investigated CXCL8 production using canine epithelial cells did not investigate the effect of TNF-α; however, similar to our study, cells stimulated with IL-4 and IFN-γ showed no effect on CXCL8 production by the buccal epithelium in vitro [15]. In the present study, in particular, CPEKs supplemented with IFN-γ showed a significant decrease in CXCL8 production compared to the vehicle. Although the mechanism through which IFN-γ may decrease IL-8 production was not investigated in this study, the findings might be associated with the decreased CXCL8 concentrations in CPEKs supplemented with the mixture of IFN-γ/TNF-α compared to those subjected to a single treatment with TNF-α.

Human keratinocytes stimulated with the mixture of IFN-γ/TNF-α showed significantly increased CCL concentrations, including CCL3, CCL4, CCL5, and most notably CCL2 [16]. In a previous study using CPEKs, TNF-α supplementations induced CCL5 production in canine keratinocytes [17]. Similarly, in this study, a single treatment with either IFN-γ or TNF-α induced significant increases in CCL2 concentrations; however, the IFN-γ/TNF-α mixture synergistically enhanced CCL2 production compared to single treatments. In addition, single treatments with IFN-γ and IL-4 did not induce IL-6 production in a previous study using canine buccal epithelium [15]. Previous studies based on human keratinocyte supplementation with the mixture of IFN-γ/TNF-α induced increased IL-6 concentrations [13,15]. The present study also revealed that single treatments with IFN-γ or TNF-α did not induce IL-6 production by canine keratinocytes but synergistic effects were observed in CPEKs supplemented with the IFN-γ/TNF-α mixture. Similarly, in a previous study based on human endothelial cells, the IFN-γ/TNF-α mixture synergistically increased the expression of both CXCL9 and CXCL10 [6]. In addition, synergistic effects of IFN-γ/TNF in the production of CCL5 have been reported in canine keratinocytes [17]. Although the detailed mechanisms underlying the synergistic effect of the mixture of IFN-γ/TNF-α were not elucidated in this study, the results suggest that this mixture might be preferably used rather than single treatments when testing cytokine stimulation in keratinocytes.

In inflammatory cutaneous reactions, IFN-γ and TNF-α are produced by T lymphocytes and play a critical role in the induction of epithelial cell activation, with their specific receptors expressed on keratinocytes [18]. In addition, the elevated expression of IFN-γ and TNF-α has also been reported in dogs with AD [10,11]. IL-6, CXCL8, and CCL2 derived from activated keratinocytes stimulated by IFN-γ and TNF-α are pro-inflammatory cytokines and chemokines, and play critical roles in recruiting and activating immune cells [19,20,21]. IL-6 plays a crucial role in the defense against invasion by infectious agents or tissue damage [19]. CXCL8 has a critical function as a chemoattractant of neutrophils, recruiting them to inflammatory sites by the trafficking of various inflammatory mediators [20]. In addition, CCL2 recruits monocytes, memory T cells, and dendritic cells in response to tissue injury or infection [21]. Therefore, the keratinocyte-derived cytokines IL-6, CXCL8, and CCL2 induced by IFN-γ and TNF-α might aggravate inflammation by activated keratinocytes. tt. In addition, it is necessary to determine whether IL6, CXCL8, and CCL2 levels are upregulated in dogs with atopic dermatitis and whether the symptoms of atopic dermatitis are alleviated when these cytokines are controlled.

In this study, no increased production of keratinocyte-derived cytokines was observed in CPEKs incubated with the Th2 cytokines IL-4 or IL-13. In a previous study involving humans, increased CCL 26 production in keratinocytes stimulated with IL-4 and IL-13 has been reported [22]. In addition, IL-4 and IL-13 stimulation on keratinocytes has been reported to downregulate the process of epidermal barrier formation in vitro [23]. Supplementation of human keratinocytes with IL-4 and IL-13 resulted in decreased expression of the epidermal barrier components keratin 1, keratin 10, desmoglein 1, and desmocollin 1 [23]. In addition, the incubation of keratinocytes with IL-4 and IL-13 significantly reduced filaggrin gene expression [24]. Furthermore, skin-equivalent models using skin-on-a-chip supplementation with IL-4 and IL-13 showed epidermal spongiosis similar to that observed in AD lesions [25]. Therefore, the effects of IL-4 and IL-13 supplementation on other cytokines or epidermal barrier formation by canine keratinocytes need to be elucidated.

Th17-type IL-17A, which is upregulated in human and canine AD, is reported to induce the production of pro-inflammatory cytokines by activated human keratinocytes [10,26]; therefore, in addition to IFN-γ and TNF-α, future studies could investigate whether IL-17A induces multiple cytokine production by activated canine keratinocytes.

Potential limitations in this study include the fact that IL-10, IL-1ß, and IL12 were barely detectable in the collected samples. Although 100 mL of the cell culture supernatant was used in each ELISA assay, no detectable peaks were observed for any of them. Therefore, it is possible that the concentrations of IL-10, IL-1β, and IL-12 in the supernatants from CPEKs were lower than the detection limit of the commercial ELISA kits used in this study. Other assays, such as the Luminex multiplex assay, can usually detect lower concentrations of cytokines. Therefore, future studies using different methods to analyze cytokine concentrations need to be conducted.

## 5. Conclusions

In conclusion, the TNF-α/IFN-γ mixture induces the production of the pro-inflammatory cytokines and chemokines IL-6, CXCL8, and CCL2 by canine keratinocytes in vitro. The Th1-type cytokines TNF-α/IFN-γ might aggravate skin inflammation through the secretion of IL-6, CXCL8, and CCL2 by activated keratinocytes. The in vitro culture system used in this study might be a useful tool to further investigate cytokine-mediated crosstalk between keratinocytes and immune cells and new therapeutic strategies for the regulation of cytokine expression in inflammatory canine skin diseases.

## Data Availability

The original contributions presented in this study are included in the article. Further inquiries can be directed to the corresponding author.
